# Efficacy and safety of TNF-alpha antagonists in children with juvenile idiopathic arthritis who started treatment under 4 years of age

**DOI:** 10.1186/1546-0096-12-S1-P339

**Published:** 2014-09-17

**Authors:** Clara Giménez Roca, Estíbaliz Iglesias, Rosa Bou, Vicente Torrente-Segarra, Judith Sánchez-Manubens, Joan Calzada-Hernández, Samuel Hernández, Sílvia Ricart, Jordi Antón

**Affiliations:** 1 Pediatric Rheumatology, Hospital Sant Joan de Deú, Esplugues del Llobregat, Spain

## Introduction

The experience in the use of tumour necrosis factor (TNF) antagonists in children below 4 years is limited, although there are some trials in the literature which support safety and efficacy under this age.

## Objectives

To assess efficacy and safety of TNF-alpha antagonists (anti-TNF) in a cohort of patients with Juvenile Idiopathic Arthritis (JIA) who began treatment under 4 years-old. Assess relapse rate after methotrexate and/or anti-TNF withdrawal.

## Methods

We made a retrospective charts review of our non-systemic JIA patients treated with anti-TNF under 4 years of age between January 2006 and April 2013. Demographics, epidemiologic, clinical, laboratory data and rate of relapse after treatment withdrawal due to clinical remission were collected. Efficacy and safety endpoints included side-effects and time to achieve clinical remission.

## Results

We included 27 patients, 23 received etanercept and 4 adalimumab with a median age of 3.01 (range 0.88-3.97) years at anti-TNF beginning and 1.94 (range 0.18-5.44) and 2.39 (range 0.18-7.24) years of treatment and follow up respectively. All patients had previously received Disease Modifying Antirheumatic Drugs at optimal dose. Nineteen patients reached clinical remission on treatment in a median time of 9.1 (range 6.23-21.17) months. Four of those relapsed during treatment. Six developed mild side-effects (22.2%): 3 primary non complicated varicella zoster virus infections, 1 pneumonia, gastrointestinal and respiratory mild infections in a patient with primary immunodeficiency and 1 case of constipation. None serious side-effects were described. Eleven patients who reached clinical remission relapsed after treatment withdrawal. None achieved clinical remission off treatment.

## Conclusion

Most patients reached clinical remission on anti-TNF. In our cohort of patients, etanercept and adalimumab were safe, with mostly mild infections and no serious side-effects. We observed a high relapse rate during treatment withdrawal.

## Disclosure of interest

None declared

